# *Clostridium difficile* appendicitis in an immunocompromised patient: a case report and review of the literature

**DOI:** 10.1186/s13256-020-02592-6

**Published:** 2021-01-05

**Authors:** Charles Dac Pham, Duong Tommy Hua

**Affiliations:** Division of Hospital Medicine, Department of Medicine, Harbor-UCLA Medical Center, 1000 West Carson Street, Torrance, CA 90502 USA

**Keywords:** *Clostridium difficile*, Appendicitis, Extraintestinal, Extracolonic

## Abstract

**Background:**

*Clostridium difficile *(*C. difficile*) is a common cause of infectious colitis in individuals with prior antibiotic or hospital exposure. Extraintestinal manifestations of *C. difficile* infections, however, are rare. Here we present a case of *C. difficile* appendicitis in an immunocompromised patient.

**Case presentation:**

A 53-year-old Caucasian male presented to the emergency room for two days of lower abdominal pain associated with nausea and subjective fevers. He otherwise denied having diarrhea or hematochezia. He did not have any recent hospitalizations, nursing home stays, or antibiotic exposure. His past medical history was notable for stage III tonsillar squamous cell carcinoma for which he was status post tonsillectomy, radiation therapy, and chemotherapy (cisplatin 4 days prior to presentation). He was afebrile with tenderness to palpation in the bilateral lower quadrants, right greater than left. His white blood cell (WBC) count was 15.6 × 10^3^ cells/μL. Computed tomography (CT) of the abdomen and pelvis showed marked edema and inflammation of the cecum and ascending colon as well as an enlarged appendix with surrounding inflammatory changes with a small amount of free fluid in the right paracolic gutter. He was treated non-surgically with antibiotics. He did not clinically improve and on hospital day 3, he developed diarrhea for which *C. difficile* stool polymerase chain reaction was sent. Repeat CT of the abdomen and pelvis was performed which showed progression to pan-colitis and persistent appendicitis. *C. difficile* testing later resulted positive, for which oral vancomycin was started. The patient markedly improved with medical management alone and was subsequently discharged on oral vancomycin.

**Conclusions:**

Our case highlights the importance of maintaining a high index of suspicion for *C. difficile* in a patient presenting with both appendicitis and colitis, with prompt diagnosis and treatment being essential.

## Background

*Clostridium difficile *(*C. difficile*) is an anaerobic, gram-positive spore-forming bacillus that is commonly found as part of the human colonic flora. It is one of the most common causes of healthcare—associated infections in the United States, with recent estimates suggesting an estimated 450,000 individuals affected annually [1]. While *C. difficile* infection (CDI) is commonly associated with colitis, extraintestinal manifestations account for just 0.17% of cases, with many of these patients having significant comorbidities, a history of prior antibiotic use, and/or concurrent intestinal involvement [2]. Given the paucity of data, the role of *C. difficile* in the pathogenesis of extraintestinal infections remains unclear. Here we present a rare case of *C. difficile* appendicitis in an immunocompromised patient.

## Case presentation

A 53-year-old Caucasian male presented to the emergency room for two days of lower abdominal pain associated with nausea, non-bilious vomiting, and subjective fevers. He otherwise denied having diarrhea, hematochezia, melena, or dysuria. He did not have any recent hospitalizations, nursing home stays, or antibiotic exposure. He denied recent travel, sick contacts, or dietary changes. His past medical history is notable for stage III tonsillar squamous cell carcinoma for which he was status post tonsillectomy, radiation therapy, and chemotherapy (cisplatin 4 days prior to presentation).

He was afebrile (36.9 °C) with a pulse of 110 beats per minute and blood pressure of 134/94 mmHg. Physical exam was notable for a non-distended abdomen, tenderness to palpation in the bilateral lower quadrants (right greater than left), and no rebound or guarding.

Laboratory examination was pertinent for a white blood cell (WBC) count of 15.6 × 10^3^ cells/μL (reference range 4.5–10 cells/μL), blood urea nitrogen (BUN) 24 mg/dL (reference range 8–20 mg/dL), creatinine of 1.35 mg/dL (reference range 0.64–1.27 mg/dL), and lactate 2.6 mmol/L (reference range 0.5–2.2 mmol/L). Computed tomography (CT) with contrast of the abdomen and pelvis showed marked edema and inflammation of the cecum and ascending colon as well as an enlarged appendix with surrounding inflammatory changes with a small amount of free fluid in the right paracolic gutter. The patient was fluid resuscitated and started on broad spectrum antibiotics with cefepime and metronidazole. Surgery was consulted due to concern for appendicitis. They deferred surgical intervention given that it was unclear if this represented colitis with secondary inflammation of the appendix rather than a direct appendiceal source. Conservative treatment with antibiotics was recommended with plans for operative management if the patient failed to improve.

**Figure 1:**
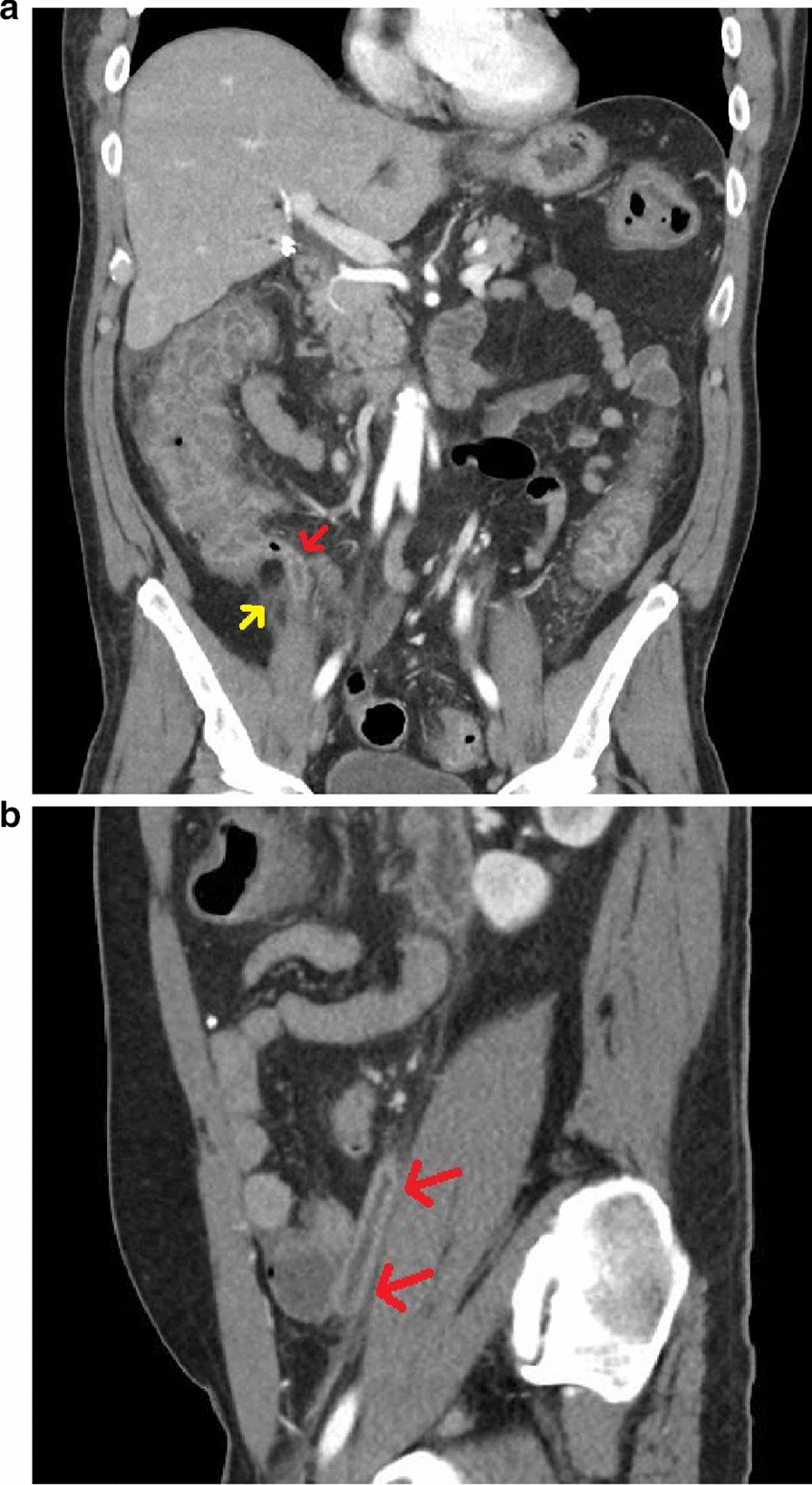
Coronal (**a**) and sagittal (**b**) CT of the abdomen and pelvis with contrast demonstrating enlargement of the appendix with wall thickening and enhancement (red arrows). Periappendiceal inflammation is also present (yellow arrow)

On hospital days 1–2, the patient continued to have significant abdominal pain, which started to localize more to the right lower quadrant. He continued to deny any diarrhea and remained afebrile. His WBC count remained elevated, but his creatinine improved after fluids. On hospital day 3, the patient started having diarrhea for which *C. difficile* testing was sent. Because the patient was not clinically improving, repeat CT of the abdomen and pelvis with contrast (Fig. 1) was performed which showed progression of his colitis, now extending from the cecum to the rectum as well as findings concerning for appendicitis as seen on the prior CT scan. *C. difficile* testing was positive for which the patient was started on oral vancomycin. On hospital days 4–6, the patient’s symptoms and exam markedly improved with normalization of his WBC count. Surgery continued to recommend non-surgical management given significant clinical improvement with antibiotics alone. The patient was discharged on hospital day 7 with oral vancomycin.

## Discussion

Extraintestinal CDI is rare and has been described in only a limited number of reports. Examples of extraintestinal manifestations that have been reported include bacteremia (with one patient having *C. difficile* isolated in an abdominal aneurysm), intra-abdominal abscess, peritonitis, cellulitis, reactive arthritis, osteomyelitis, brain abscess, and empyema [2, 3]. Acute appendicitis is another one of the rare extracolonic manifestation of CDI as we have described here in our case. From review of the literature, there have been very few reported cases of *C. difficile* appendicitis [4-7].

*C. difficile* has generally been known as a pathogen responsible for colitis, particularly in the setting of prior antibiotic or hospital exposure. Recent chemotherapy is also another important risk factor [8], which was the case in our patient as he was actively undergoing treatment for his tonsillar cancer. It has been hypothesized that the disruption of the gut microbiota allows for the proliferation of toxigenic *C. difficile* and subsequent infection. *C. difficile* colitis is a process mediated by bacterial release of Toxins A and B, whose role is to inactivate members of the Rho family of guanosine triphosphatases (GTPases), which leads to colonocyte death, inflammation, and loss of the intestinal epithelial barrier function [9]. The pathogenesis of extraintestinal CDI, however, remains unclear. However, given his concurrent pancolitis, it is likely that direct spread of *C. difficile* from the intestinal lumen resulted in his appendicitis. This is in opposition to *C. difficile* isolated from distant sites described in other case series, where it is thought that bacterium enters through blood circulation [2].

Two possible mechanisms for *C. difficile* appendicitis have been suggested: direct infection from the toxin itself versus obstruction of the appendiceal lumen from the adjacent colitis [5]. It is plausible that both of these processes play a role, as the luminal obstruction can lead to ischemia and compromise of the mucosal barrier, resulting in bacterial invasion of the appendix by intraluminal bacteria [10]. When this occurs, a mix of aerobic and anaerobic organisms, particularly *Escherichia coli* and *Bacteroides* spp., are typically implicated [11], as opposed to *C. difficile* which is exceedingly uncommon as detailed in our case.

We should also note that the role of *C. difficile* in causing extraintestinal infections have been questioned. One case series showed that many isolates were either part of polymicrobial flora or intra-abdominal fluid collections near the colon in the setting of recent fecal spillage [3]. Furthermore, it has been found that not all extraintestinal *C. difficile* isolates produce toxins [3], which is essential in the pathogenesis of *C. difficile* colitis as discussed above (therefore, culture, while the most sensitive test for *C. difficile* infection [8], has largely been replaced by methods that detect toxigenic strains). While it is certainly possible that the patient’s *C. difficile* colitis (as confirmed by stool PCR testing) and his appendicitis represented distinct and unrelated processes, the clinical presentation, radiographic features, and overwhelming response to *C. difficile* directed antibiotic therapy suggests that *C. difficile* was the most likely underlying etiology.

Given the rarity of *C. difficile* appendicitis, no specific treatment guidelines exist. We performed a literature review to explore the demographics, management, and outcomes of those with *C. difficile* appendicitis (see Table 1). Using PubMed, we searched for articles with both keywords of “clostridium difficile” and “appendicitis,” which yielded 25 results. We reviewed each individual article further to ensure that the case report was of appendicitis that was attributable to *C. difficile*. We reviewed a total of six cases of *C. difficile* appendicitis and found that none of the patients were immunocompromised. Interestingly, five out of the six cases of *C. difficile* appendicitis involved patients over 50 years-old. All six patients initially survived; however, one had a recurrent episode leading to septic shock and death. Three patients eventually underwent surgical intervention, two of which survived. Management was not specified in two of the cases. As described above, we, in conjunction with our surgical colleagues, opted for medical therapy alone given the patient’s significant clinical improvement. We should note, however, that given the limited number of reported cases, further studies on optimal treatment is still needed.

**Table 1:** Literature review of cases of *C. difficile* appendicitis

Author (Year)	Age (yo)	Gender	Immunocompromised	Co-Morbidities	Treatment	Outcome
Garcia-Lechuz *et al*. (2001)	53	Male	No	Hypothyroidism	Imipenem Appendectomy	Survived
Brown *et al*. (2007)	72	Male	No	COPD	Metronidazole Appendectomy	Survived
Mattila *et al*. (2013)	97	Female	Unknown	Breast cancer Dementia	Unknown	Survived
Mattila *et al*. (2013)	59	Female	No	Epilepsy	Unknown	Survived
Ridha *et al*. (2017)	30	Male	No	None	Metronidazole Levofloxacin Oral vancomycin	Survived
Coyne *et al*. (1997)	76	Female	No	ESRD on HD	1st episode: Metronidazole 2nd episode (4 weeks later): proctocolectomy	1st episode: Survived 2nd episode: death from septic shock

## Conclusions

Acute appendicitis is a rare extraintestinal manifestation of CDI. While the exact pathogenesis remains unclear, its significance cannot be overlooked. Our case highlights the importance of maintaining a high index of suspicion for *C. difficile* in a patient presenting with both appendicitis and colitis, as prompt diagnosis and treatment are essential.

## Data Availability

Not applicable.

## Abbreviations

*C. difficile*: Clostridium difficile
WBC: White blood cell
CT: Computed tomography
BUN: Blood urea nitrogen
CDI: *C. difficile* Infection
